# Network pharmacology-based study on the mechanism of Yiganling capsule in hepatitis B treatment

**DOI:** 10.1186/s12906-020-2815-y

**Published:** 2020-02-05

**Authors:** Chao Lu, Wanjin Fu, Renpeng Zhou, Wei Hu

**Affiliations:** Department of Clinical Pharmacology, The Second Hospital of Anhui Medical University, Hefei, 230601 China

**Keywords:** Network pharmacology, Hepatitis B, Yiganling capsule, Clusters, Signaling pathway

## Abstract

**Background:**

Yiganling (YGL) capsule is a traditional Chinese medicine preparation consisting of eight herbs that has been clinically proven to have a favorable treatment effect on Hepatitis B (HB). However, due to its multiple targets and multi-pharmacological effects, the mechanisms of YGL capsule in the treatment of HB are unknown.

**Methods:**

First, the chemical constituents of YGL capsules were obtained from the Chinese medicine database, and YGL capsules were constructed. Second, active compounds were screened by the ADME model. The target fishing model was used to screen the corresponding targets of active compounds and to construct a compounds and compound targets network. Using human disease databases and literature mining, we systematically identified genes associated with HB, constructed disease-specific protein-protein interaction networks, and performed clustering and enrichment analyses of these networks. These networks were then merged to obtain a compound-disease target network, and cluster and enrichment analyses were performed on the compound-disease target network to acquire a compounds-disease targets-mechanism network and a clustering network.

**Results:**

We successfully built eight pharmacological network diagrams, including four primary networks and other network maps. The four dominating network maps included a HB disease-associated protein-protein interaction network, a YGL capsule compounds-target network, a YGL capsule ingredient target-HB disease target network, and a YGL-HB disease mechanism network. Other networks included a pathway of HB disease targets, the HB disease protein-protein interaction cluster analysis network, and the YGL-HB target clustering network.

**Conclusion:**

This study successfully forecasted, illuminated, and confirmed the synergistic effects of HB disease molecules and discovered the potential of HB relevant targets, clusters, and target-related biological processes and signaling pathways. Our research not only provides theoretical support for the molecular and pharmacological mechanisms of YGL capsule in HB treatment, but also provides new research methods for the study of the other traditional Chinese medicinal compounds.

## Background

Hepatitis B (HB) is a chronic disease caused by hepatitis b virus (HBV) infection. HBV can cause liver inflammation, liver tissue fibrosis, cirrhosis, and even liver cancer [1]. HB is now the world’s largest public health problem, with more than 200 million people worldwide suffering from HBV, and causing the death of 600,000 people each year. The incidence of HB in China is relatively high, accounting for approximately 33% of worldwide cases. With 78 million chronic carriers and approximately 300,000 deaths each year from HBV-related diseases, HB is an urgent public health challenge in China [2].

China has accumulated a lot of experience in the prevention and treatment of HB. In addition to the HB vaccine in to prevent HBV infection and the use of anti-disease drugs to treat HB disease, other Chinese medicinal compounds are widely used in clinical practice because they have a good rate of improving the rate of HB patients and reducing the clinical symptoms of HB [3].

Yiganling (YGL) capsule is a drug approved by the China Food and Drug Administration (CFDA) for the treatment of HB disease. The drug has strong anti-inflammatory activity, improves liver function, a choleretic and hepatic protective effect, decreases transaminase function, and increases the rate of HB patients. YGL capsules contain eight Chinese herbal medicines, including Radix Rhei Et Rhizome (RRER), Paeoniae Radix Alba (PRA), Radix Bupleuri (RB), Artemisiae Scopariae Herba (ASH), Radix Bupleuri Fortunes Bossfern Rhizome (RBFBR), licorice, *Panax ginseng* C. A. Mey (PGCAM), and *Hedysarum multijugum* Maxim (HMM) [4]. YGL is clinically used in combination with antiviral drugs to treat HB and can improve the rate of negative conversion. However, due to limitations in research techniques and economic considerations, research into the pharmacodynamics of traditional Chinese medicines (TCM) has been primarily focused on single targets or a few pathways [5, 6]. It is difficult to reveal the synergy between multi-component and multiple targets in disease treatment. Currently, the precise mechanisms of YGL capsule in the treatment of HB are unknown.

The research on TCM prescriptions has been using the research method of chemical drugs for a long time, by combining the separation of chemical components of TCM and simple screening of active compounds to study action mechanism. Although this method can discover some of the pharmacological substance basis and mechanism of action of TCM prescriptions, it is difficult to really interpret the overall mechanism of TCM action, due to TCM prescription has the characteristics of multi-component multi-target synergy and indication function diversity [7, 8]. These restrictions have limited the promotion and use of Chinese medicine internationally.

**Fig. 1:**
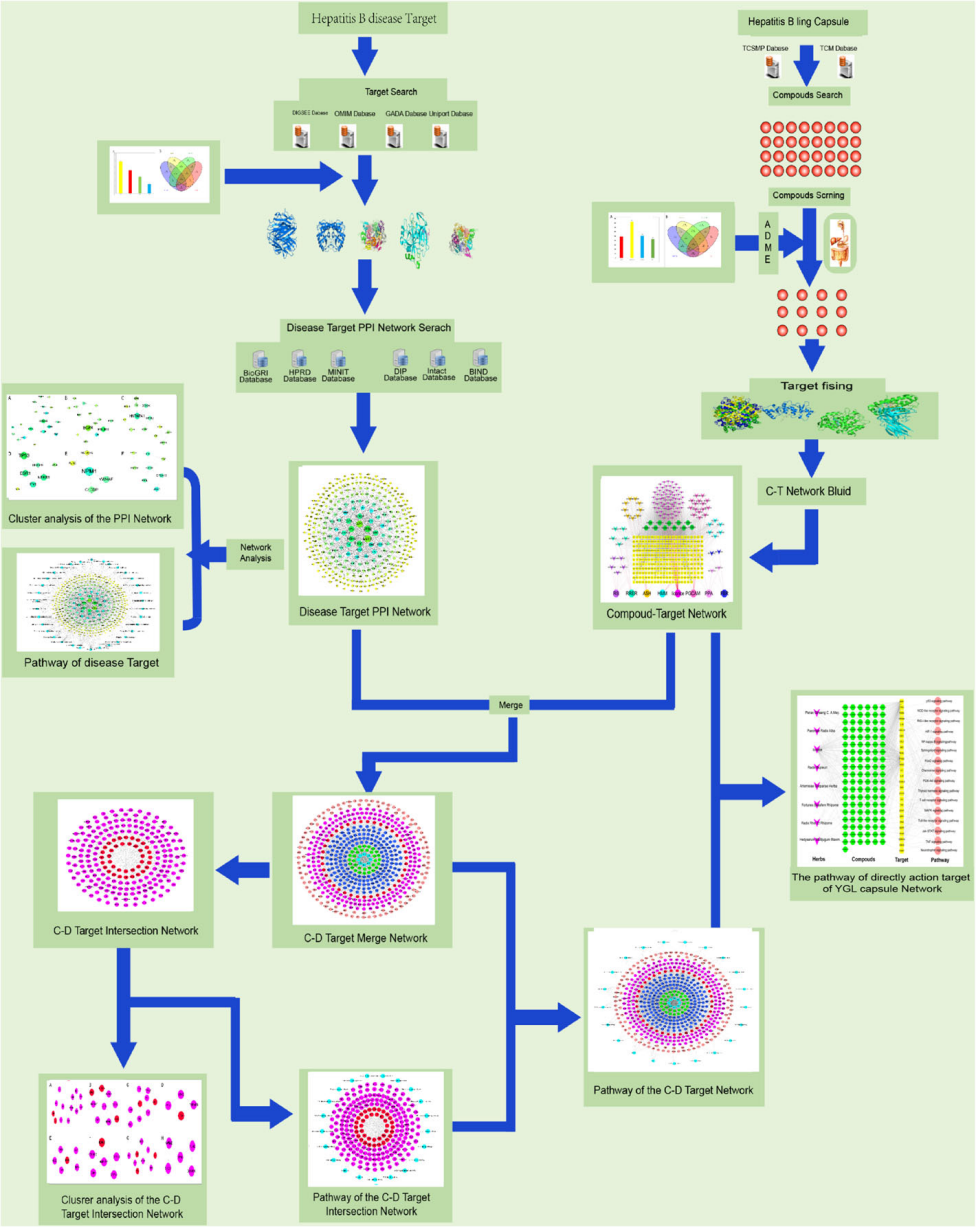
YGL Capsule treatment of HB network pharmacological flow chart

Network pharmacology is an emerging discipline that has been developed in recent years through the integration of bioinformatics and pharmacology. Through the establishment of a database of medicinal chemical components, researchers can now find relationship between components and targets and the relationship between targets and target diseases to systematically mine existing biological data and abstract those data into a network relationship model that systematically explains the role of a drug or drugs in disease treatment [9, 10]. To provide evidence for the clinical application of YGL capsule in HB treatment, we used network pharmacy to study multi-component and multi-target drugs, exploring chemical constituents, target sites, and action pathways. The flowchart of using network pharmacology methods to study the mechanism of action of YGL capsule in HB treatment is shown in Fig. 1.

## Methods

### Data preparation

#### Construction of the chemical constituents database of the YGL capsule

The Latin names of the eight herbs in the YGL capsule were put into the TCM database (http://tcm.cmu.edu.tw/) and the TCM Systems Pharmacology Database (TCMSP, http://tcmspw.com/) to retrieve the chemical composition of each herb and their corresponding pharmacodynamic parameters. TCM database is an earlier database of traditional Chinese medicine small molecules, which contains 443 Chinese medicines and more than 20,000 compounds. The database classifies all Chinese medicines and provides a simple query interface. The TCMSP Database analysis platform is a comprehensive Chinese medicine analysis platform. For the first time, it includes all Chinese medicine ingredients in the Chinese Pharmacopoeia, including 502 Chinese medicines and 29,394 compounds. The database provides 12 ADME parameters for each compound for screening of potential active compounds and prediction of targets, as well as enrichment information for target pathways and disease-related targets. In addition, these two databases also provide a functional interface for compound target prediction, which can facilitate the prediction of targets for new compounds. The retrieved results were combined to construct a chemical composition database of YGL capsule [11, 12].

#### ADME screening of the YGL capsule

To screen the active ingredients in YGL capsules more efficiently and economically, we used the ADME (absorption, distribution, metabolism, and excretion) models to screen the active chemical components. The ADME model uses in vitro experiments combined with in silico technology to study the in vivo processing of compounds, which changes the status of traditional pharmacokinetics based on animal experiments, and achieves high throughput, efficiency, and accuracy [13, 14]. This has greatly improved research in drug development. The four parameters oral bioavailability (OB), Caco-2, drug-like (DL), and half-life (HL) indicators were used to virtually screen potential active compounds of each herb in the YGL capsule.

OB refers to the relative amount of drug absorbed into systemic blood circulation after administration by the extravascular route and reflects whether the compound has a key indicator of intrinsic activity. Many compounds fail to produce therapeutic effects due to the lack of kinetic characteristics (especially oral bioavailability) and are unable to reach in vivo targets [15]. The OB values were obtained on a bioinformatics system using the OBioavail 1.1 model, which calculates the relationship between the composition and the P4503A4 enzyme and transporter (p-glycoprotein). An OB value stands for the capacity of the compound to enter the circulatory system in the human body [16]. We termed compounds with OB > 30% as potentially active ingredients [17].

Caco-2 permeability refers to the permeability of a drug in intestinal epithelial cells, which determines its absorption in the intestine. The human intestinal cell line Caco-2 is usually used to study the passive diffusion of drugs in the intestinal epithelium. The transport rate (nm/s) of our Caco-2 monolayer component represents the epithelial permeability of the drug in the intestine [18]. Our study used Caco-2 > − 0.4 as the cutoff for potentially active ingredients [17].

DL values are evaluated by calculating the resemblance between a compound and a certified medicine structure. Calculating the DL value of a compound early in drug development is helpful in the screening of excellent compounds and increases the rate of new drug development. This study established a similar evaluation model for the YGL capsule component database using the Tanimoto parameters for the evaluation model:

$$ \mathrm{T}\left(\mathrm{x},\mathrm{y}\right)=\frac{x\cdotp y}{{\left|x\right|}^2+{\left|y\right|}^2-x\cdotp y} $$

where x is a descriptor for a compound calculated using DRAGON software (http://www.talete.mi.it/products/dragon_description.htm) and y is all drugs in the DrugBank database (https://www.drugbank.ca/) average descriptor [19]. We used DL > 0.18 as the cutoff for potentially active ingredients [17].

HL is the most important pharmacokinetic parameter of a drug. It refers to the time taken to reduce the blood concentration of the compound into the body by half. The rate of elimination of the drug in the body is often used to calculate the administration time, guide rational application of the drug, and to determine a dosage regimen [20, 21] . We used HL > 3 h as the cutoff for potentially active ingredients.

#### Predicting targets of YGL capsules

We imported the selected active chemical components of YGL capsules into the Traditional Chinese Medicine Systems Pharmacology Database to find the relationship between each chemical component and their potential targets. The database obtains drug-target interactions mainly through the following two aspects: (1) Drug-targets that have been experimentally validated from the HIT database, (2) For the target combination of drugs without experimental data support, the SysDT models were mainly used for prediction [12]. Obtaining a target includes multiple species targets and requires a more concise expression of the relationship between the active ingredient and the target. Thus, we used the UniProt database (http://www.uniprot.org/) to retrieve the gene name of all targets, with the selected species being human.

#### Searching targets of HB diseases

We gathered HB disease-related targets from four sources: (1) UniProt (https://www.uniprot.org/); (2) Online Mendelian Inheritance in Man (OMIM) (http://omim.org/); (3) DIGSEE (http://210.107.182.61/geneSearch/);(4) Genetic Association Database (GAD) (https://geneticassociationdb.nih.gov/). In these human disease target databases, our study used the keyword “Hepatitis B” and species “homo species” to search for targets related to HB [22] . The disease targets retrieved from the above databases were pooled to obtain targets related to HB. Targets of HB disease in different databases were placed in the Venny 2.1 system (http://bioinfogp.cnb.csic.es/tools/venny/index.html) to obtain targets associated with HB.

#### Protein-protein interaction (PPI) data

This study used the bisogenet 3.0 plugin for Cytoscape software to find the relationship between target proteins in HB. Bisogenet directly accesses and integrates data from six published biological protein-protein interaction databases to obtain more complete data on PPIs. First, we entered the Bisogenet plug-in for HB-related proteins, and then set the Bisogenet plug-in parameters. The main settings are as follows: First we set the “Organism” option to “*Homo sapiens*” in the identifiers settings; second, we set the “Biorelation types” in the Data Setting to “DIP”, “BIOGRID”, “HPRD”, “INTACT”, “MINT”, and “BIND “; third, we selected “Method” set to “Input nodes only”; and fourth, we set the output settings “Set Represent network nodes in term of” option to “Genes” [9, 22]. Data of all self-interacting proteins were deleted to show PPI directly and concisely.

### Network construction

#### Method for network construction

To better demonstrate the mechanism of action of YGL capsule in HB treatment, we constructed the following four dominating networks: (1) Disease PPI network; (2) Drug compounds and compound targets network (C-T network); (3) Drug compound-disease target network (C-D network); (4) Drug compounds-disease targets-mechanism Network (C-D-M Network).

These four networks were built using the open source freeware Cytoscape (version 3.7.1; https://cytoscape.org/download.html) [23]. Cytoscape is an open source software platform that graphically displays, analyzes, and edits the network. The software commonly uses bioinformatics to analyze interactions between molecules and proteins and the biological pathways of proteins. Additionally, the software contains a large number of network analysis plug-ins, which can help analyze the relationships between multi-component and multiple targets, and can obtain key nodes of the network; thus, it can help identify the primary mechanism of TCM treatment in different diseases [24, 25].

#### Cluster analysis

Some target proteins in cellular biological activities are closely related and have the same or analogous functions; these target proteins can be considered a cluster. Proteins of the same cluster are generally considered to play a synergistic role in disease progression. The topology module, biological function module, and disease module have a unified meaning in the network, and the biological function module is cognate to the topology module. Thus, the disease can be regarded as the interference and destruction of the functional model. We use the MECODE plug-in to perform cluster analysis on protein targets in complex bioinformatics networks [26, 27].

### Enrichment analysis

The C-D-M network and the cluster analysis protein were placed in the DAVID 6.8 (https://david.ncifcrf.gov/tools.jsp) database for GO enrichment and pathway enrichment analyses. Enrichment analysis results of the study were screened at *p < 0.05* and excluded specific disease pathways [28].

#### Molecular docking verification

To verify the accuracy of the TCSMP database on molecular prediction targets, this study used AutoDock Vina software for molecular docking validation [29]. We selected the ESR1 target with the highest degree in the C-D-M network for verification, and 106 molecules interacted with the target in this study. Fistly,to see how the compound interacts directly with the target, we used LigPlot software to create a two-dimensional map of the compound with the ERS1 target [30]. Secondly, from the docking energy perspective, the ESR1 inhibitor SR16234 drug (InChIKey: VOHOCSJONOJOSD-SCIDSJFVSA-N) from the TTD database was selected as a positive group. Finally, the three-dimensional structure after docking the remaining unverified molecules with the target was observed by using PyMol software [31].

## Results

### Drug C-T network analysis

#### Database of YGL capsule ingredients

There are 1070 unique compounds in the YGL capsule database, including ACH with 53 compounds, FBR with 31 compounds, HMM with 87 compounds, licorice with 280 compounds, PRA with 85 compounds, PGC with 190 compounds, RB with 349 compounds, and RRER with 92 compounds (Table 1). Detailed information on the chemical constituents of each herb and the chemical parameters of the compounds in YGL capsule are described in Additional file 1: Table S1.

**Table 1:** The number of compounds in each herb of YGL capsules

Herbal Name	Compounds Number
ASH	53
FBR	31
HMM	87
Licorice	280
PRA	85
PGCA	190
RB	349
RRER	92

#### Active compounds screening result

**Fig. 2:**
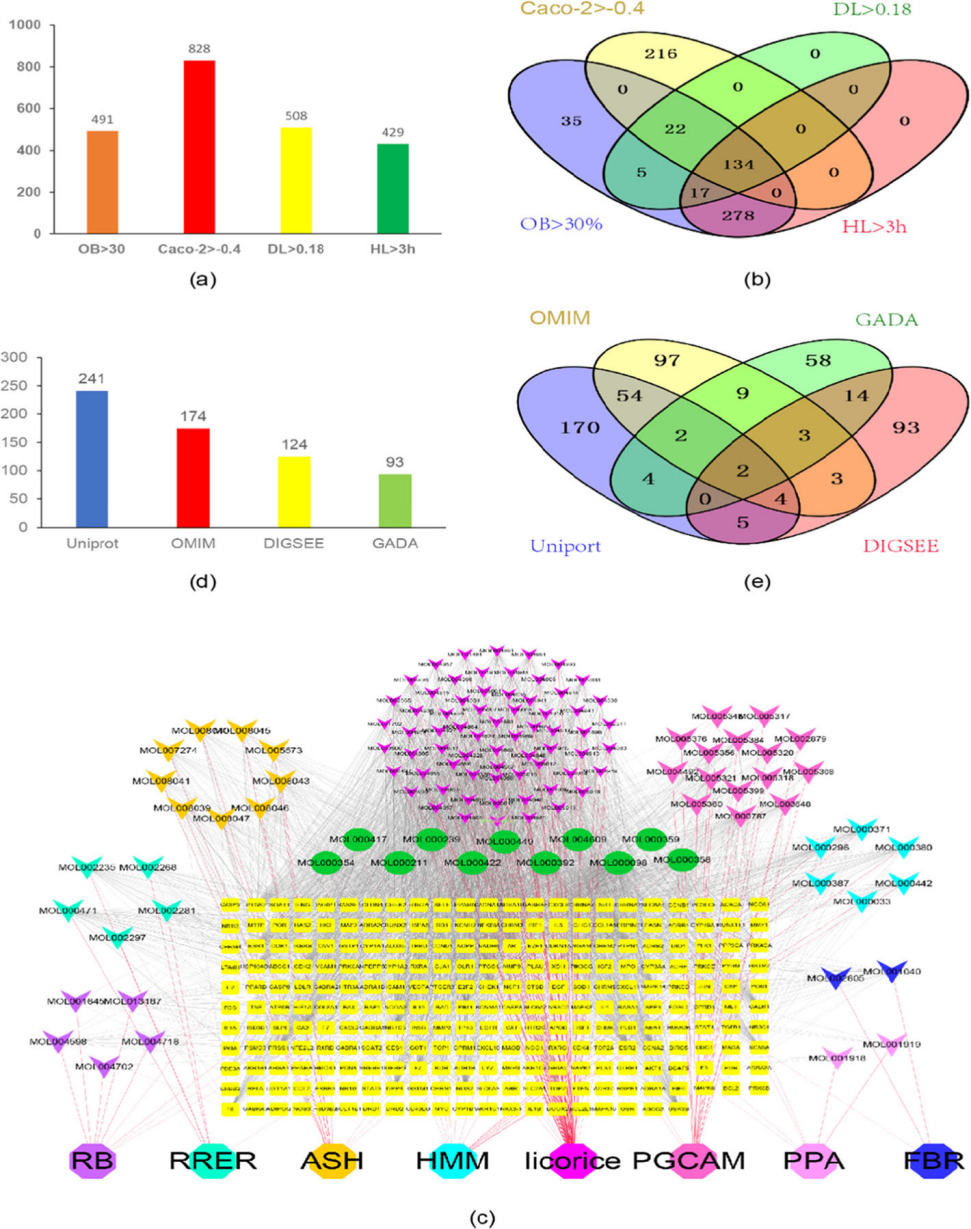
AMDE screening of active compounds in YGL capsule, the YGL capsule compound-compound target network, and HB disease target mining. **a** Active compounds according to the OB, CACO-2, DL, and HL screening results. **b** Venn diagram of the active compounds according to the four indicator values (OB, CACO-2, DL, and HL). **c** The YGL capsule compound target network consisted of 250 compound targets and 122 compounds (stars represent compounds, yellow squares represent targets). **d** The retrieval results of HB targets for different disease databases. **e** Venn diagram of the retrieval results of HB targets of different disease databases

The active compounds in the YGL capsule component database were screened using the ADME model, in which 491 compounds were obtained from OB indicators, 828 compounds were screened using Caco-2, 508 compounds were obtained from the DL index, and 429 compounds were acquired from the HL index. A total of 134 potentially active compounds were obtained by intersecting the above results (Fig. 2a and b). Detailed information on the screening of active ingredients in YGL capsules is described in Additional file 2: Tables S2–S6.

### Drug compound-compound target network

Twelve of the 134 potentially active compounds screened by the ADME model were removed because they were not predicted to interact with human targets. The established YGL capsule C-T network consisted of 380 nodes and 2832 edges, including the eight herbs contained in YGL capsules, 122 active compounds, and 250 targets constitute the network node. These potentially active ingredients produced a total of 2688 interactions with targets. The number of active ingredients and targets of the eight herbs that were contained in the YGL Chinese medicine compound in the C-D network are as follows: RRER contains six potentially active ingredients that act on 77 targets; PRA includes six potentially active compounds that correspond to 95 targets; RB contains 10 potentially active ingredients and 191 targets; ASH includes 13 potentially active ingredients with 185 targets; Licorice contains 75 potentially active ingredients with 230 targets; PGCA includes 18 potentially active compounds that correspond to 117 targets; HMM contains 13 potentially active ingredients with 206 targets; and FBR includes three potentially active ingredients that correspond to 82 targets (Table 2).

**Table 2:** Number of potentially active compounds per herb and the number of targets

Herbal name	Potential active compound number	Targets Number
ASH	13	85
FBR	3	82
HMM	13	206
licorice	75	230
PRA	6	95
PGCA	18	11
RB	10	191
RRER	6	77

In this C-T network, the interactions of the nodes were not in equilibrium. Some compounds can regulate multiple targets (for example, quercetin compounds can act on 161 potential targets, kaempferol can regulate 72 targets, and aringenin can affect 47 potential targets). Conversely, some ingredients can only affect a few targets, for instance, Chrysanthemaxanthin, which only affects ESR1 protein. Similarly, some targets can be synergistically coordinated by multiple chemical components, while others are only affected by a few compounds. For example, the F2 target can be regulated by 131 compounds, and NOS3 protein can be regulated by 111 compounds, but some proteins such as RXRG and UGT1A1 are regulated by only one compound. The uneven distribution of compounds-target network nodes indicated that YGL capsule treatment may rely on the role of some key compounds. The network details are described in Fig. 2c and Additional file 3: Tables S7–S8.

### Disease target network analysis

#### HB disease-related targets

From the UniProt database, we obtained 241 HB-related proteins, 174 from the OMIM database, 93 protein from the GAD database, and 124 from the DIGSEE system. A total of 594 HB disease-related proteins were obtained (Fig. 2d and e). Network details are provided in Additional file 4: Table S9. Database information of PPI relationships are described in Additional file 5: Table S10.

#### HB disease PPI network

We deleted self-interacting proteins and non-interacting proteins to obtain a HB PPI network. The PPI network consisted of 332 nodes and 1201 edges. The size of the node area in the network is positively correlated with the magnitude of its degree in the PPI network. The degree of each node is closely related to other important features in the network, the higher the degree of the protein targets in the network the more likely it is to play an important role in disease development. The protein nodes such as EGFR, APP, GRB2, TP53, PIK3R1, IKBKG, ESR1, and NFKB1 have higher degrees in the PPI network, correspondingly, they presented a larger region in the network. These eight protein targets corresponded to a degree of 64, 63, 58, 54, 52, 50, 46, and 45. Additionally, these protein targets may be closely linked to HB development. Network details are described in Additional file 15: Figure S1 and Additional file 6: Tables S11–S12.

#### Clusters analysis of the HB disease PPI network

The PPI network was clustered by cluster analysis using the MCODE package and six clusters were used to analyze the mechanism of HB disease (see Table 3 for each type of clustering target, and Additional file 15: Figure S2 for clustering target relationship). By analyzing the relevant biological processes, molecular functions, and cellular components of every cluster, we found that HB disease was primarily resistant to viruses, innate immunity, congenital immunity, cellular immunity, cell migration, cell proliferation, apoptosis, and other processes. Details of each cluster enrichment analysis are described in Additional file 7: Table S13.

**Table 3:** HB disease PPI network cluster analysis

Cluster	Score	Nodes	Edges	Node
1	5.556	19	50	HCK, SRC, PLCG1, IKBKE, PSMB9, PSMA3, PSMA7, CHUK, AIRE, IKBKB, PSMB8, PSMB5, FASLG, IKBKG, MET, TNF, AR, TNFAIP3, PSMD1
2	4	15	28	HLA-E, HLA-B, HLA-C, CD8A, PTK2B, KRT18, KRT8, EGFR, CXCR4, IFNAR1, FYN, PIK3R1, SOCS1, CCR5, HLA-A
3	3.333	13	20	SRPK2, CEBPB, MOV10, IGF2BP1, CEBPA, CD81, HNRNPA1, HIST1H1E, ICAM1, DDX3X, NOLC1, VDR, SRPK1
4	3	7	9	CUL5, KRT19, NFKB1, ESR1, TP53, FN1, HNRNPK
5	3	5	6	YWHAE, NPM1, GLTSCR2, C1QBP, PUM3
6	2.857	8	10	VIM, CDK1, BECN1, EIF2AK2, GFAP, PML, JAK1, HMGB1

#### The pathway of the HB network

**Fig. 3:**
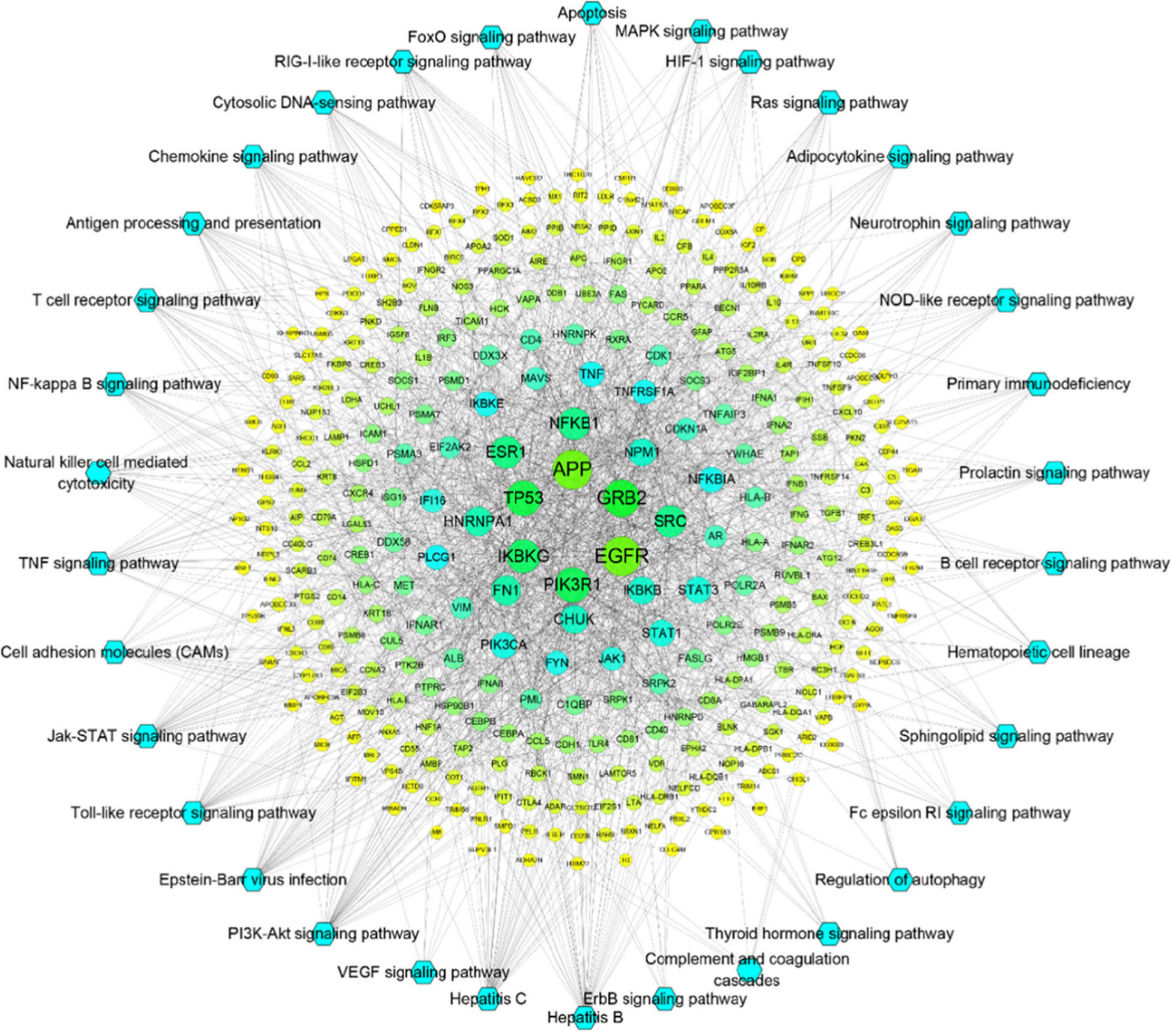
Pathways of Hepatitis B disease targets

HB disease-related proteins were put into DAVID for pathway analysis, and 34 pathways were acquired after deleting results for specific disease pathways. The Hepatitis C and HB pathways contain 41 and 40 proteins, respectively. The PI3K-Akt signaling pathway contains 39 proteins, the Epstein-Barr virus infection contains 32 protein, 28 proteins were enriched in the Toll-like receptor pathway, 27 proteins were enriched into the JAK/STAT pathway, cell adhesion molecules (CAMs) contains 24 protein, and the TNF pathway contains 24 proteins. The study obtained a pathway of the HB network by aggregating the disease PPI network and HB-related protein pathway analysis. The network directly revealed interactions between disease proteins and the relationship of disease-related pathways (Fig. 3). The network consists of 228 nodes (34 pathways and 332 disease-related proteins) and 1806 edges. Detailed information of the HB disease pathway is provided in Additional file 8: Tables S14–S15.

### YGL capsule-HB network analysis

#### YGL capsule-HB disease target network

**Fig. 4:**
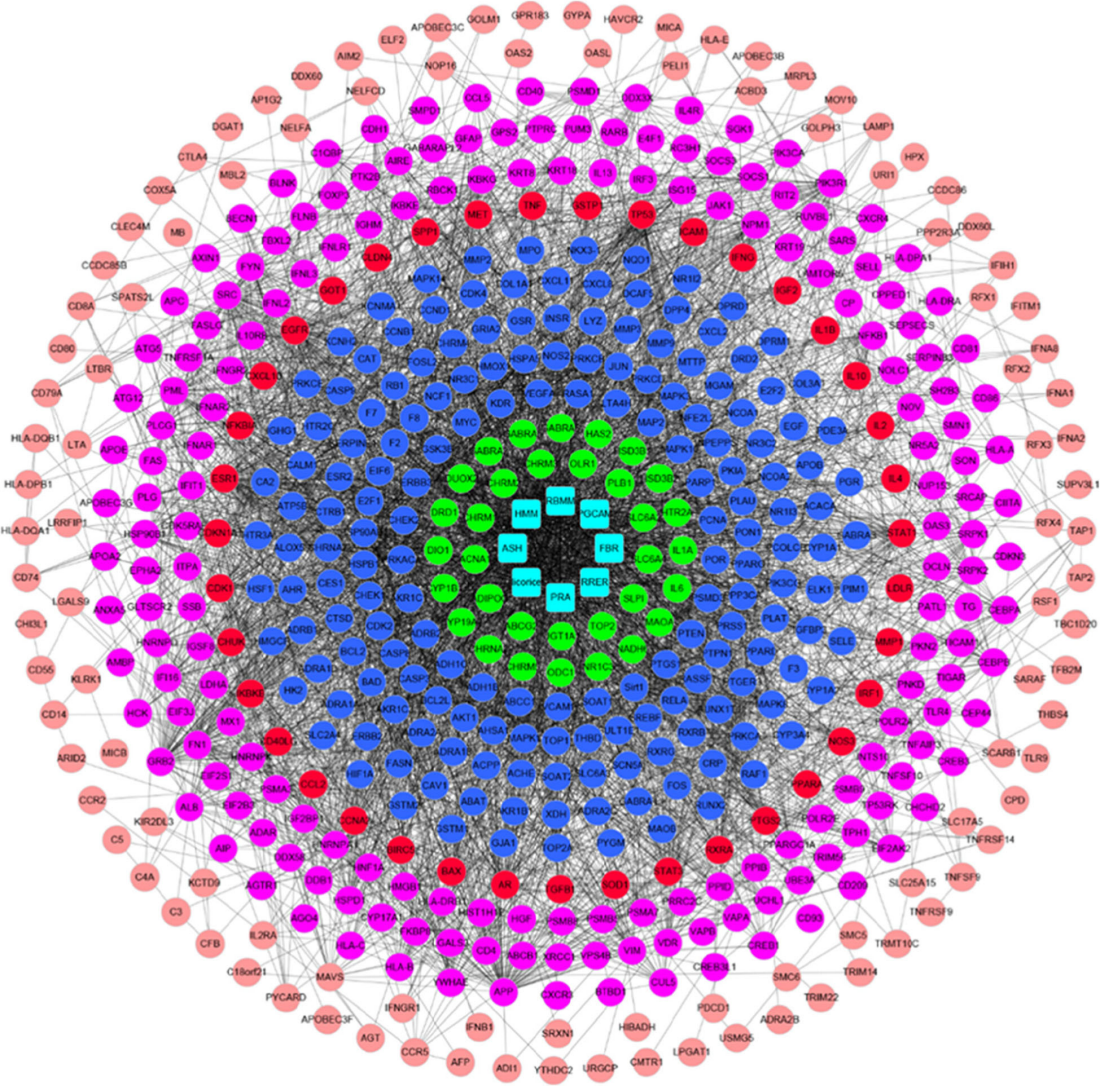
YGL capsule-HB disease network (blue squares represent herbal medicines, green circles represent targets not associated with HB disease targets, blue circles represent compounds indirectly related to HB targets, red circles represent compounds that directly affect HB targets, purple circles represent indirect YGL targets, and brown circles represent targets not associated with YGL capsule targets)

The YGL capsule-HB disease C-D network was acquire by combining the C-T network with the disease PPI network. To show the correspondence between components and targets in a simple and intuitive way, relationships between herbs and their components were hidden. The C-D network succinctly demonstrates the interaction between each active compound in the YGL capsule and HB disease targets, and multi-component multiple target interactions are presented in a network map. The C-D network is composed of 551 nodes, and each node has 4106 interactions. According to the interaction between the active compounds and HB disease targets, 551 nodes in the C-D network were divided into six categories using different shapes, patterns, and colors for the nodes. The classification of the C-D network from the inside to the outside is as follows: the first type is the eight herbal name nodes contained in the YGL capsule; the second type is the 26 nodes that were independent of the disease target and only interacted with the active compound; the third type contained 178 active component targets that were indirectly related to disease targets; the fourth type had 39 active component targets that were directly related to disease targets; the fifth category had 182 nodes that were active components of disease targets that had an indirect effect on the disease targets; the sixth category had 110 nodes that were disease targets that had an effect on disease targets by the active ingredient. The C-D network is shown in Fig. 4 and the node interactions of the network are provided in Additional file 9: Tables S16–S17.

**Fig. 5:**
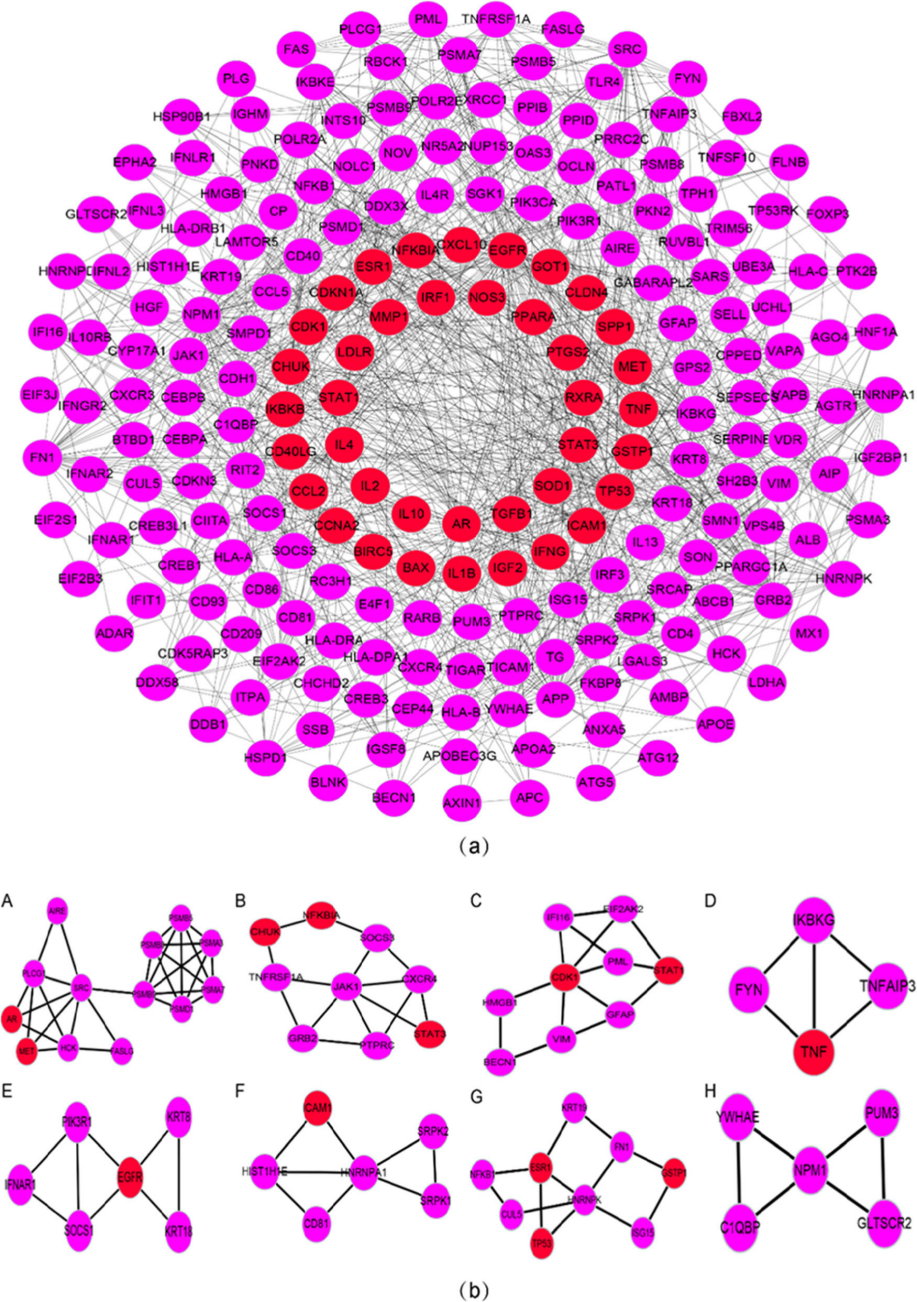
**a** YGL capsule-HB disease network that only includes disease targets (red circles represent compounds that directly affect HB targets, purple circles represent compound with an indirect role in HB targets). **b** Clusters of the YGL compound-HB network (**a-h** represent clusters 1–8)

To directly analyze the targets of YGL capsule active ingredients in HB, we first removed the herbal name node from the C-D network, and then removed the node that had no interaction between the disease target and the active component target, which yielded the YGL capsule-HB disease network only containing disease targets. The network was composed of 222 nodes and 908 edges and is shown in Fig. 5; node interactions of the network are provided in Additional file 10: Tables S18–S19. We first clustered the network and then analyzed pathways in the enrichment analysis for each cluster to obtain the primary pathway of YGL capsule treatment in HB (Table 4).

**Table 4:** Clusters within the YGL capsule-HB network

Cluster	Score	Nodes	Edges	Node
1	4.833	13	29	AIRE, PSMB9, HCK, PSMA7, PSMB5, PSMA3, PSMB8, AR, PLCG1, MET, FASLG, SRC, PSMD1
2	3.5	9	14	CHUK, SOCS3, STAT3, GRB2, CXCR4, NFKBIA, TNFRSF1A, JAK1, PTPRC
3	3.5	9	14	GFAP, BECN1, VIM, EIF2AK2, PML, HMGB1, IFI16, CDK1, STAT1
4	3.333	4	5	TNFAIP3, IKBKG, FYN, TNF
5	3.2	6	8	KRT8, EGFR, KRT18, PIK3R1, SOCS1, IFNAR1
6	3.2	6	8	CD81, ICAM1, HNRNPA1, SRPK1, SRPK2, HIST1H1E
7	3	9	12	KRT19, GSTP1, NFKB1, ESR1, FN1, CUL5, ISG15, HNRNPK,TP53
8	3	5	6	GLTSCR2, C1QBP, NPM1, YWHAE, PUM3

#### Clusters within the YGL capsule-HB target network

The YGL capsule-HB target network clustering analysis used the same method as the PPI network clustering analysis to obtain eight different clusters. The main mechanism of the effect of YGL capsule in HB was obtained by analyzing the enrichment of each cluster. The enrichment analysis results are shown in Fig. 5b and the details are provided in Additional file 11: Table S20.

Through the analysis of network enrichment, we found that YGL capsule can treat HB through activities including anti-viral, regulating immunity, regulating metabolism, regulating cell proliferation, apoptosis, and migration. The main antiviral effect of YGL capsules was by regulating viral entry into host cells, viral genome replication, and defense responses to viruses and viral processes. The regulation of immune function by YGL capsule mainly included activation of innate immune responses, inflammatory responses, and by regulating interferon-gamma, innate immune responses and tumor necrosis factor. The metabolic effects of YGL capsule were: cellular response to amino acid stimulus, cellular response to cytokine stimulus, cellular response to glucose starvation, cellular response to lipopolysaccharide, and cellular response to organic cyclic compounds.

### The YGL capsule C-D-M network

The direct and indirect action targets of YGL capsule were placed in the DAVID database to analyze action pathways. Specific disease pathways were deleted from the results, leaving 21 pathways. The relationships between these proteins and pathways were placed in Cytoscape to construct the pathway of the YGL-HB network. Details of the enrichment analysis results are provided in Additional file 15: Figure S3 and Additional file 12: Tables S21–S22.

**Fig. 6:**
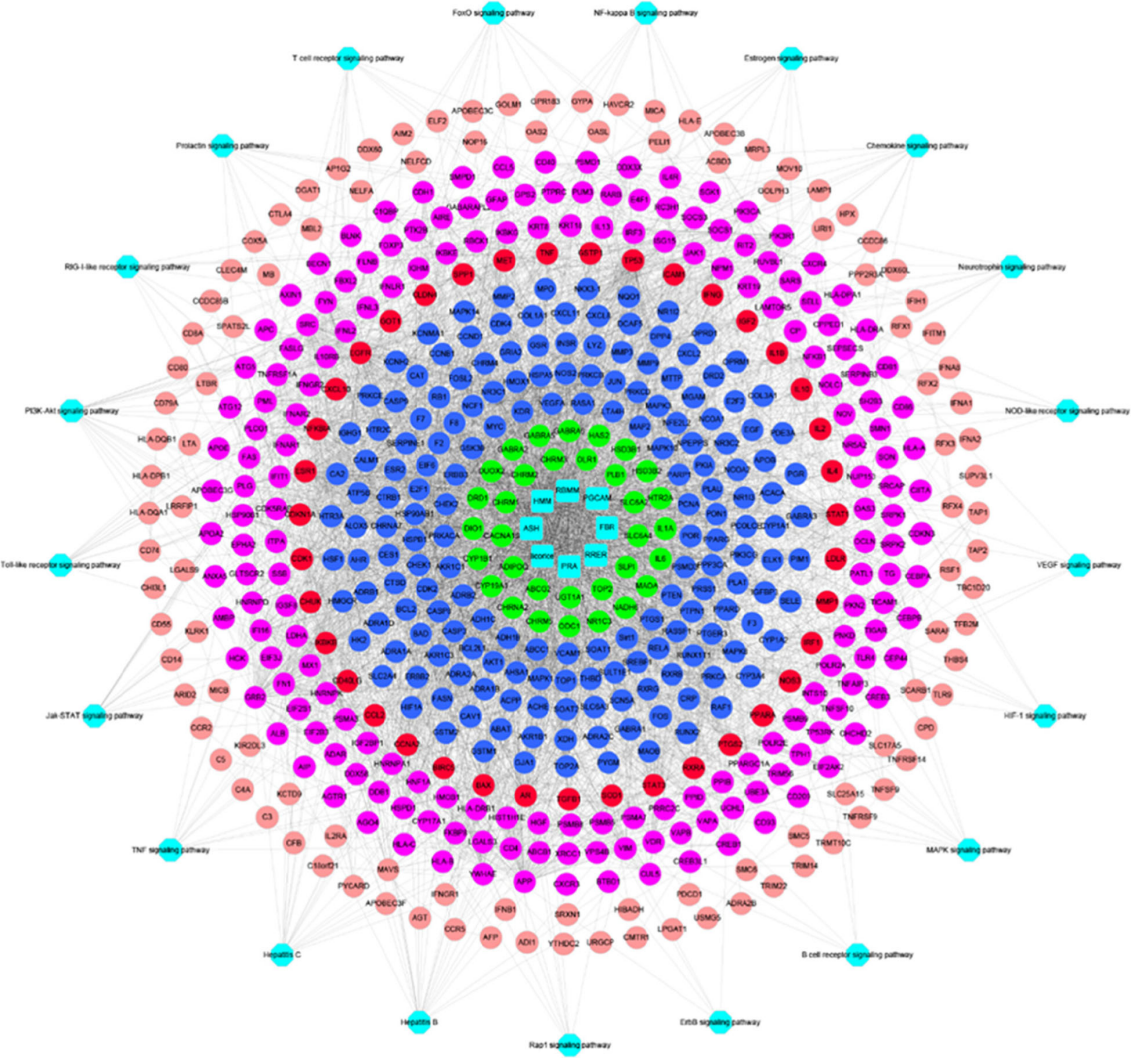
Pathways of the YGL capsule-Hepatitis B network only including intersection targets (blue squares represent herbal medicines, green circles represent targets not associated with Hepatitis B disease, blue circles represent compounds that have an indirect role in Hepatitis B targets, red circles represent compounds that directly affect Hepatitis B targets, purple circles represent YGL capsule compounds that have indirect targets, brown circles represent those that have nothing to do with YGL capsule targets, and blue hexagons represent the enriched pathways)

Pathways of the YGL capsule-HB network only include intersecting target networks that were obtained by merging the pathway of the YGL capsule-HB network with the YGL capsule-HB disease targets network. This network consisted of 571 nodes, and the relationship between the nodes produced 4338 edges. Detailed information is provided in Fig. 6 and Additional file 13: Tables S23–S24. The network consisted of 72 HB disease-associated proteins mapped by YGL compounds and 21 pathways derived from protein enrichment. In these 21 pathways, the PI3K-Akt pathway was enriched by 21 proteins and had the highest degree of enrichment; the JAK/STAT pathway was enriched by 15 proteins; the TNF pathway was enriched by 14 proteins; Toll-like receptor signaling was enriched by 13 proteins; chemokine signaling was enriched by 12 proteins; the T cell receptor and FoxO signaling pathways were enriched by 11 proteins; the RIG-I-like receptor and MAPK signaling pathways were enriched by 10 proteins; the NF-κB, estrogen, and neurotrophin pathways were enriched by nine proteins. These pathways may represent the primary mechanisms for how YGL capsule ameliorates HB disease.

**Fig. 7:**
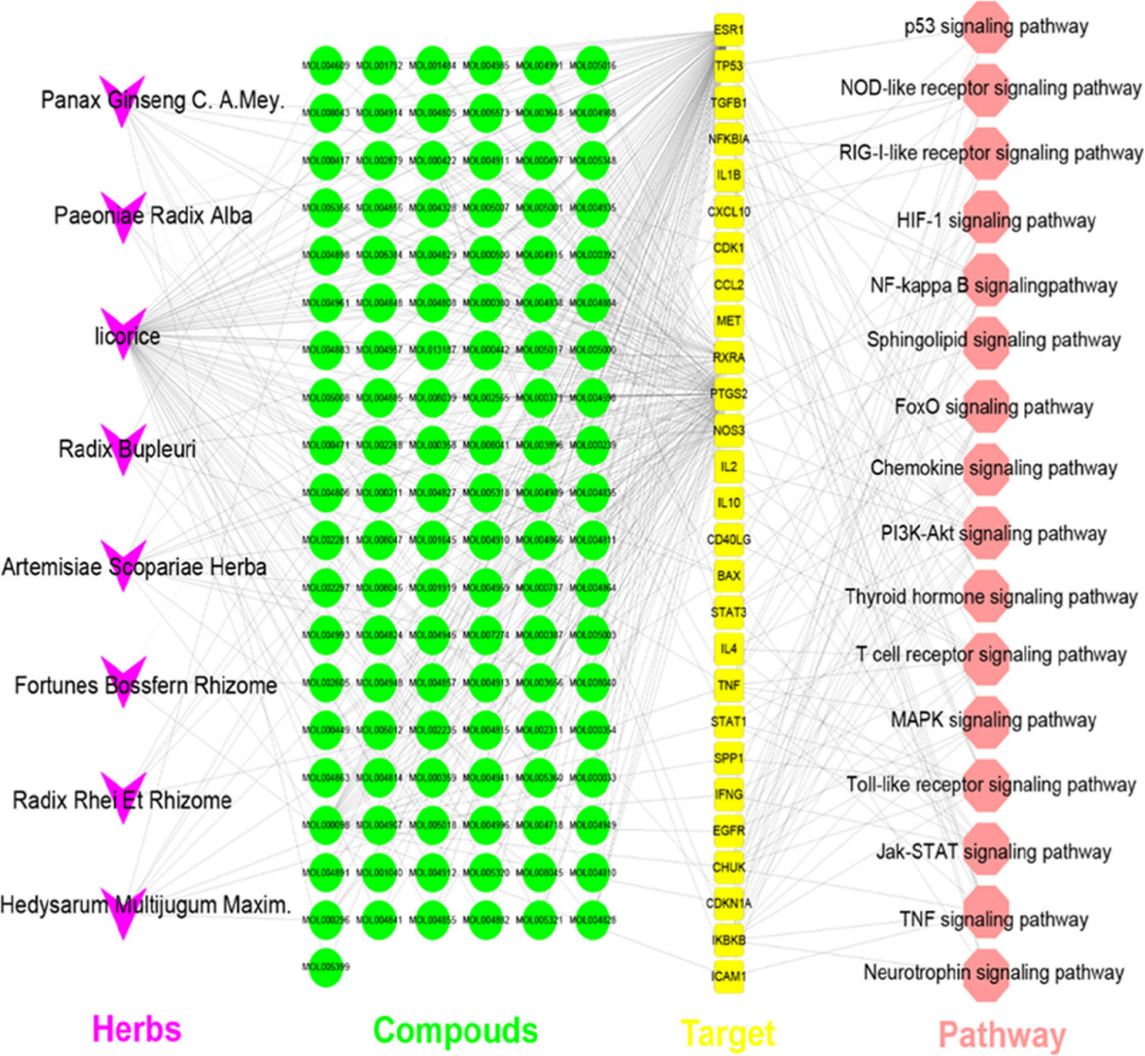
The pathways that were found to be direct action targets of YGL capsules (magenta polygons represent herbs, green circles are compounds, yellow squares are targets, and the red/brown hexagons are signaling pathways)

The C-D-M network could clearly define relationships between herbs, targets, and targets and pathways, but lacked the ability to demonstrate relationships between herbs and compounds. Additionally, due to the large number of nodes and the relationships between the networks, the network is still relatively complicated. For example, licorice can act on 289 indirect targets and 38 direct targets. These targets can be enriched into 21 pathways. To succinctly show the main mechanism of action of YGL capsule, we only retained direct targets and carried out enrichment analysis on the direct targets. These results are merged with the Fig. 2 network to obtain a pathway of direct action targets of YGL capsule. The network consisted of eight herbs, 116 compounds, 27 targets, and 16 pathways, and displayed four types of targets in different regions. Detailed information is provided in Fig. 7 and Additional file 14: Tables S25–S26.

### Molecular docking verification

**Fig. 8:**
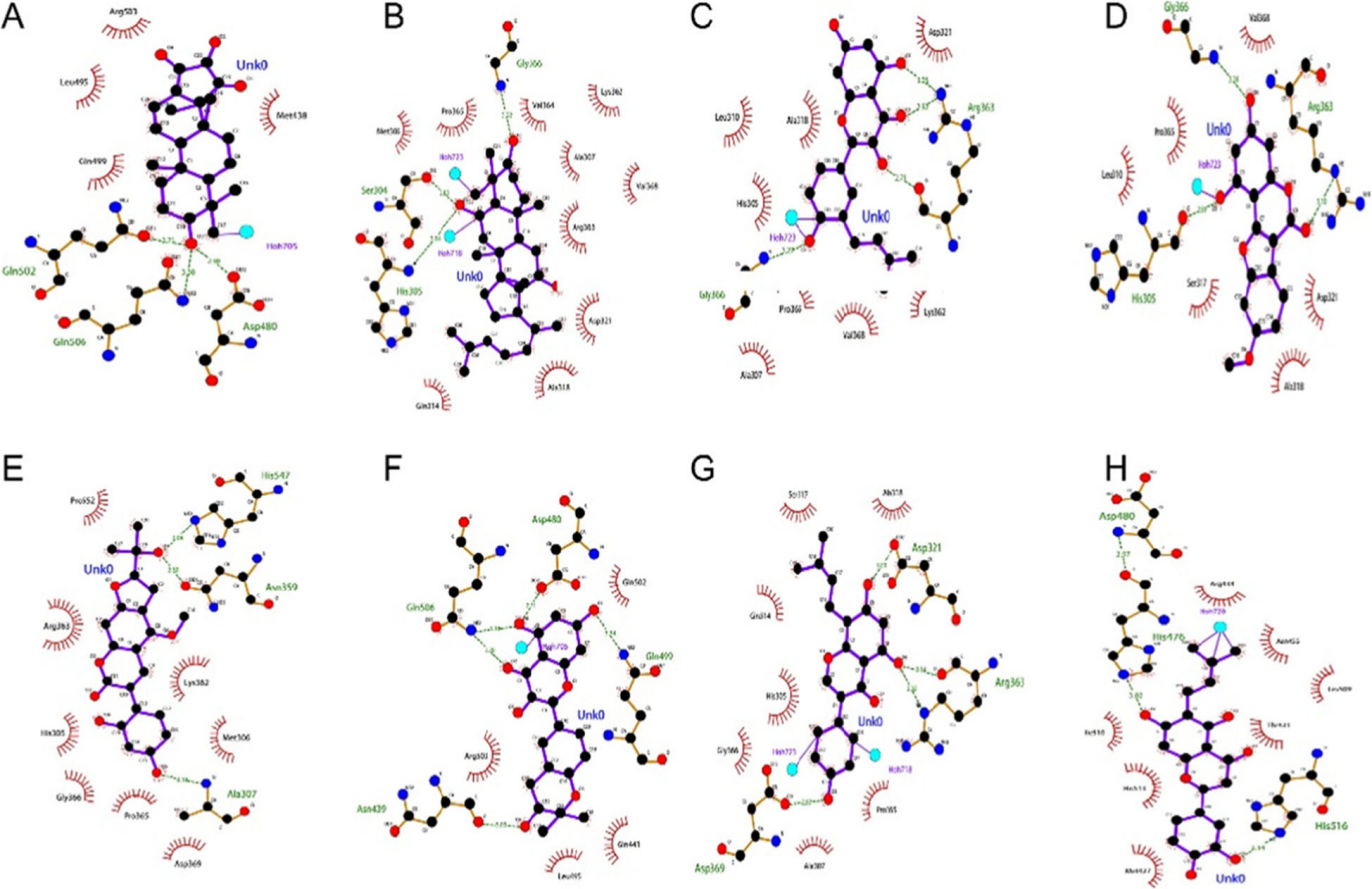
Molecular and ERS1 target part docking results(**a** represents the docking result of MOL005384 with the target, **b** represents the docking result of MOL00727 with the target, **c** represents the docking result of MOL000471 with the target, **d** represents the docking result of MOL000787 with the target, **e** represents the docking result of MOL001792 with the target, **f** represents the docking result of MOL002235 with the target, **g** represents the docking result of MOL002268 with the target, **h** represents the docking result of MOL000354 with the target)

By observing the two-dimensional structure of the compound after docking with the target, it was found that 91 molecules directly interact with the target by hydrogen bonding. Since the energy of the positive drug after docking is − 7.6 kcal / mol, the lower the energy after docking, the more stable the conformation after docking, the docking energy was chosen to be less than or equal to − 7.6 kcal/mol conformation. Among the remaining 15 molecules, MOL005003, MOL004912 and MOL004866 molecules were selected. By observing the three-dimensional structure of the molecule and the target, the MOL000392 and MOL002297 compounds can have a π-π interaction with the benzene ring of the target through the benzene ring of the molecule. Part of the docking results are shown in Fig. 8. Through the above three methods, we verified that 96 compounds have direct interaction with the ESR1 target, and 90.56% of the compounds interact with the ERS1 target. The TCMSP database was preliminarily verified using the above method to predict compound target stability and reliability.

## Discussion

Hepatocyte damage from HB disease is not directly caused by HBV, but is caused by HBV-mediated host cell immunity [32]. In this study, through cluster and enrichment analyses of HB disease targets, many protein targets in the target participate in anti-inflammatory responses and immune processes. Some protein targets (e.g.PIK3CA, NFKB1, IKBKB, CHUK, IKBKG, TNF, and GRB2) act on more than 10 pathways, most of which are involved in inflammatory responses, innate immunity, and secondary immune processes. By regulating key proteins in these pathways, these may be targets for future HB treatment.

HB as an acute and chronic infectious disease caused by HBV infection that can cause systemic metabolic disorders, abnormal digestion and absorption, jaundice, nervous system diseases, immune disorders, renal insufficiency, and other systemic phenotypes [33]. These symptoms may be related to the in vivo biological effects of pathways associated with HB-related target enrichment. The pathways enriched by PPI targets of HB disease mainly included the following 23 pathways: PI3K-Akt, TNF, T cell receptor, Epstein-Barr virus infection, Toll-like receptor signaling, JAK/STAT, natural killer T cell-mediated cytotoxicity, NF-κB, RIG-I-like receptor signaling, VEGF, antigen processing and presentation, MAPK, FoxO, Ras, HIF-1, primary immunodeficiency, NOD-like receptor signaling, neurotrophin, adipocytokine, sphingolipid signaling, hematopoietic cell lineages, B cell receptor signaling, and chemokine signaling. These pathways are involved in processes such as antiviral, primary immune response, cellular immunity, cytokine release, cell migration, angiogenesis, and hepatocyte energy metabolism.

Analysis of the C-D-M network revealed that 16 pathways were associated with YGL treatment of HB disease. These pathways have been widely reported in previous studies of HB disease. In this study, nine representative pathways were selected to discuss the relationship between YGL capsules for HB treatment and these pathways.

The PI3K-Akt pathway can be activated by a variety of different stimuli and toxins and regulates many basic cellular processes, such as cell growth, transcription, translation, cell division and proliferation, migration, apoptosis, and glycogen metabolism [34–36]. The PI3K-Akt signaling pathway also plays an important role in regulating HBV-DNA replication in infected hepatocytes [37]. Akt activation can regulate HBV-RNA transcription and reduce HBV-DNA replication in hepatocytes. Treatment of HBV-positive hepatocytes with PI3K inhibitors or the mTOR inhibitor rapamycin increased the transcription of 3.5 KB and 2.4 KB viral RNA, thereby inhibiting HBV-DNA replication [38].

The JAK/STAT pathway is common to many cytokines, is widely involved in cell proliferation, differentiation, apoptosis, and inflammation, and can interact with other signaling pathways through negative regulators [39]. The X-gene product of HBV (HBx) leads to activation of the Jak1/STAT pathway and enhances hepatocyte proliferation by activating the Jak1-tyrosine kinase. HBx-mediated JAK/STAT activation may be associated with various pathogenic phenomena caused by HBV infection. For instance, constitutive STAT3 activation leads to increased interleukin-6 expression, which is the main mediator of the acute phase of HB and HB-associated cirrhosis [40].

TNF protein is expressed by activated Kupffer cells, NK cells, T cells, B cells, mast cells, and monocytes in the liver. TNF plays a crucial role in inflammatory responses and immune regulation, and is also involved in cell division, proliferation, and apoptosis. TNF activates neutrophils and monocytes by signaling the presence of infected tissues and activates these inflammatory cells to combat and destroy pathogens [41]. However, excessive amounts of TNF may cause a rapid drop in blood pressure, even heart shock, by reducing smooth muscle and myocardial contractility. TNF is critical for HBV clearance in chronic HB (CHB) patients. As shown by in vitro experiments, TNF first inhibits viral entry into hepatocytes. Second, TNF relieves HB by promoting the release of IFN-γ by NK cells and T cells, inhibiting viral replication and destroying HBcAg signaling and its downstream effectors. Low level TNF causes weakened T cell responses, resulting in incomplete HBV clearance [42].

After HBV infection, chemokines bind to their receptors, causing chemotaxis of specific inflammatory and immune cells to the lesion, which participate in inflammatory responses. Thus, chemokines have a considerable role in the acute and chronic phase of HB [43]. For example, CXCL10 (IP-10) and its main chemotactic target cells, including T cells, B cells, monocytes/macrophages, NK cells, basophils, granulocytes, and dendritic cells are critical for the chemotaxis of various inflammatory cells into the liver to clear the virus, while also secreting inflammatory transmitters that mediate inflammatory reactions, resulting in degeneration and necrosis of infected liver cells, producing liver tissue damage. Studies have shown that patients with CHB who are treated with interferon, have decreased CXCL10 expression and viral inhibition [44]. RANTES is regulated by the expression and secretion of activated T cells and is regulated by cytokines such as IL-1, IFN-γ, and TNF-α in liver tissues of CHB patients. RANTES can also be expressed by NK cells, T cells, hepatocytes, fibroblasts, and platelet cells. RANTES expression in CHB patients is higher than in healthy people; furthermore, RANTES expression in hepatocytes is also appreciably increased in areas with severe inflammatory reactions [45].

Toll-like receptor signaling is part of the body’s natural immunity, which directly mediates natural immune responses through the recognition of pathogen-associated molecular patterns. The cascade of signals leads to the production of cytokines and synergistic stimulating factors. Toll-like receptors are the main receptors for host non-specific immunity and play a crucial role in natural and acquired immunity [46]. The dsRNA produced during HBV replication can bind to TLR3. Activation of the TLR-like pathway is associated with reduced viral replication and virus clearance. TLR3 is activated at the transcriptional level by interferon regulatory factors 3 and 7 (IRF3, IRF7). IFN-β expression also regulates initiate antiviral immunity, but can cause liver damage and necrosis [47].

IKBKB, CHUK, and NFKBIA are important genes in the NF-κB pathway that are involved in regulating various proteins involved in physiological and pathological processes such as inflammatory translation, immunity, cell proliferation, differentiation, and apoptosis. If NF-κB is overactivated, it will cause immune liver damage and promote the development of liver fibrosis by inhibiting apoptosis of hepatic stellate cells [48]. This study indicated that IKBKB is regulated by the MOL000422 compound, which acts on 11 pathways. CHUK is regulated by MOL000098, which acts on 10 pathways. NFKBIA is regulated by MOL000098 and can act on 8 pathways. The pathways associated with NFKBIA enrichment are chemokine signaling, NOD-like receptor signaling, Toll-like receptor signaling, NF-κB, T cell receptor signaling, RIG-I-like receptor signaling, TNF, and neurotrophin signaling.

The T cell receptor signaling pathway plays an absolutely central part in regulating the adaptive immune system. T lymphocytes are the main cell population of the adaptive immune system and exert immune efficacy [49]. The main cause of persistent HBV infection is due to the low specific immune function of T cells. Acute self-limiting HBV infection causes a strong virus-specific T cell response, whereas it shows a weak virus-specific T cell response in CHB patients.

In the peripheral blood of patients with CHB, the proportion of CD25+, CD8+, and Treg cells is seriously imbalanced. The proportion of CD4+, CD25+, and Treg cells is negatively correlated with the number of CD8+ T cells and positively correlated with levels of HBV replication. In early-stage HBV-associated acute liver injury, the proportion of CD4+ T cells, CD25+ T cells, and Treg cells decreases rapidly, suggesting that the immune tolerance phase of HBV persistent infection is broken. Thus, the immune system begins to stress clear hepatocytes infected with HBV, inducing pathological processes of acute liver injury [50, 51]. Our study found that the T cell signaling pathway could be regulated by nine proteins, including CD40LG, CHUK, IFNG, IKBKB, IL10, IL2, IL4, NFKBIA, and TNF.

Thyroid hormone is secreted by the thyroid gland and is divided into T3 and T4, which play an important part in regulating human development, metabolism, and nervous system activity. The levels of thyroid T3 and T4 in patients with CHB are lower than in healthy people, and thyroid levels gradually decrease with disease severity. When thyroid hormone levels are dysregulated, abnormal metabolism of proteins, fats, and sugars in the body, as well as other diseases will occur [52, 53]. Our study found that HB could affect the thyroid hormone signaling through four proteins, ESR1, RXRA, STAT1, and TP53.

YGL capsule can also directly regulate eight other pathways by acting on different targets, namely Toll-like receptor signaling, Sphingolipid signaling, RIG-I-like receptor signaling, p53, NOD-like receptor signaling, neurotrophin signaling, MAPK, HIF-1, and FoxO. Therefore, we analyzed the mechanisms of YGL capsule in HB disease treatment by applying multiple active ingredients to multiple targets, and these multiple targets were applied to the effective pathways.

## Conclusions

Our study found that some ingredients of YGL capsules can resist HBV infection by regulating the immune system and inflammatory factors, while others can affect HB by altering hormone pathways in the body that affect metabolism. We first constructed a pharmacology network by collecting the chemical constituents and chemical properties of each herbal compound, screening potential active compounds with the ADME model, virtually screening the corresponding targets of the compounds, and mining the relevant targets of HB diseases from different disease databases. Network maps to predict, mine, explore, and validate the synergy between YGL in the treatment of various compounds and targets of HB disease were also constructed. Through the construction of a PPI network of HB disease, the interaction relationship of various disease targets was revealed. Additionally, we performed cluster analysis on YGL-HB targets and successfully performed enrichment analysis of each category to obtain biological functions related to target proteins by analyzing their molecular functions, cellular components, and related pathways. Finally, we constructed an herbal-compound-direct target-pathway network and analyzed the network to reveal and predict the main biological information and pharmacological mechanisms of YGL treatment of HB. Our research allowed us to better predict the main mechanisms of YGL in HB treatment through network pharmacology. However, due to the lack of research on the concentration of active substances and the dose-effect relationship of these compounds, the specific effects of treatment are still insufficient. In view of the relationship between the concentration of the active substance of the compound and the dose-response, the therapeutic effect will be affected. In future research, we will first measure the concentration of active compounds in YGL drugs by liquid mass spectrometry, and use an in vitro model to observe the different concentrations of the effective active component against Effects of HBV protein synthesis and DNA replication. Finally, a mouse model of hepatitis B will be used to observe the pharmacodynamics and pharmacokinetic characteristics of different doses of effective active ingredients in the treatment of hepatitis B.

## Supplementary information

**Additional file 1: Table S1.** Composite compounds of YGL capsule.

**Additional file 2: Table S2.** Compounds with an oral bioavailability value greater than 30%. **Table S3.** Compounds with a Caco-2 value > − 0.4. **Table S4.** Compounds with a drug-likeness value > 0.18. **Table S5.** Compound with a half-life > 3 h . **Table S6.** The potentially bioactive compounds of YGL capsule using the ADME in silico integrative model.

**Additional file 3: Table S7.** Prediction of putative targets for compositive compounds YGL capsule. **Table S8.** Four topological feature values of targets in target-compositive compounds network.

**Additional file 4: Table S9.** The therapeutic target data for HB treatment from four resources, including Uniprot, OMIM, DIGSEE, and GADA.

**Additional file 5: Table S10.** Detailed information on six existing protein-protein interaction databases.

**Additional file 6: Table S11.** Hepatitis B disease protein- protein interaction network disease PPI Network. **Table S12.** Four topological feature values of Hepatitis B disease PPI network.

**Additional file 7: Table S13.** The enrichment analysis of the target cluster analysis of Hepatitis B disease PPI network.

**Additional file 8: Table S14.** Pathway of Hepatitis B. **Table S15.** Four topological feature values of Pathway with Hepatitis B target.

**Additional file 9: Table S16.** YGL capsule- Hepatitis B Disease target. **Table S17.** Four topological feature values of YGL capsule- Hepatitis B Disease target.

**Additional file 10: Table S18.** YGL capsule - Hepatitis B disease network only includes targets for disease. **Table S19.** Four topological feature values of YGL capsule - Hepatitis B disease network only includes targets for disease.

**Additional file 11: Table S20.** The results of target enrichment analysis were obtained after cluster analysis of YGL capsule-hepatitis B disease PPI.

**Additional file 12: Table S21.** Pathway of YGL capsule- Hepatitis B Disease target. **Table S22.** Four topological feature values of Pathway of YGL capsule- Hepatitis B Disease target.

**Additional file 13: Table S23.** Pathway for YGL capsule-HB-mechanism network only includes intersection targets network. **Table S24.** Four topological feature values of Pathway for YGL capsule-HB-mechanism network only include intersection targets network.

**Additional file 14: Table S25.** The direct action pathway targets of YGL capsule. **Table S26.** Four topological feature values of the pathways of direct action targets of YGL capsule.

**Additional file 15: Figure S1.** Hepatitis B disease target protein-protein interaction network. **Figure S2.** Results of cluster analysis for Hepatitis B disease target protein-protein interaction network. **Figure S3.** Pathways of YGL-Hepatitis B.

## Data Availability

All data are included in this manuscript and in the additional files. Datasets supporting the conclusions of this article are available as a public database from TCMSP, TCM Database@Taiwan, GACD, DIGSEE, UniPort, BioGRID, HPRD, MINT, DIP, Intact, BINDSuperPred, TTD, and OMIM.

## Abbreviations

ASH: Artemisiae Scopariae Herba
CFDA: China Food and Drug Administration
CHB: Chronic Hepatitis B
DL: Drug-Like
GADA: Genetic Association Database
HB: Hepatitis B
HBV: Hepatitis B Virus
HBx: X-gene product of the HBV
HL: Half-Life
HMM: *Hedysarum multijugum* Maxim
IRF3: Interferon regulatory factors 7
IRF7: Interferon regulatory factors 4
OB: Oral Bioavailability
OMIM: Online Mendelian Inheritance
PGCAM: *Panax ginseng* C. A. Mey
PPI: Protein–Protein Interaction
PRA: Paeoniae Radix Alba
RB: Radix Bupleuri
RBFBR: Radix Bupleuri Fortunes Bossfern Rhizome
RRER: Radix Rhei Et Rhizome
TCM: Traditional Chinese medicines
YGL: Yiganling
