# Supplementary material for: Network pharmacology-based study on the mechanism of Yiganling capsule in hepatitis B treatment
Source: BMC Complement Med Ther. 2020 Feb 5;20:37. doi: 10.1186/s12906-020-2815-y (PMC7076828; doi:10.1186/s12906-020-2815-y)
Supplement: Supplementary file 15 — Additional file 15: Figure S1. Hepatitis B disease target protein-protein interaction network. Figure S2. Results of cluster analysis for Hepatitis B disease target protein-protein interaction network. Figure S3. Pathways of YGL-Hepatitis B. [file 12906_2020_2815_MOESM15_ESM.docx]

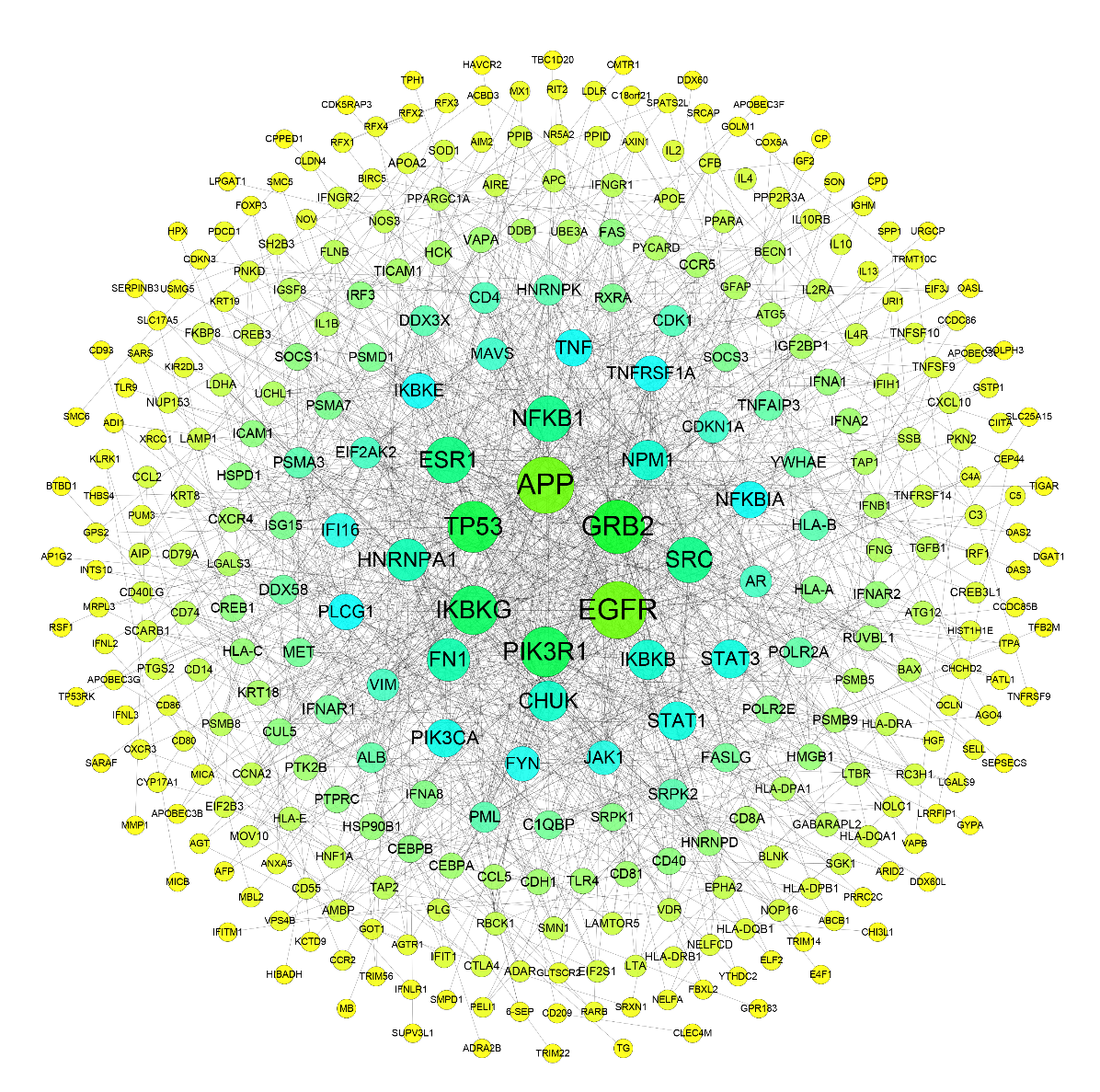


Figure S1. Hepatitis B disease target PPI Network.


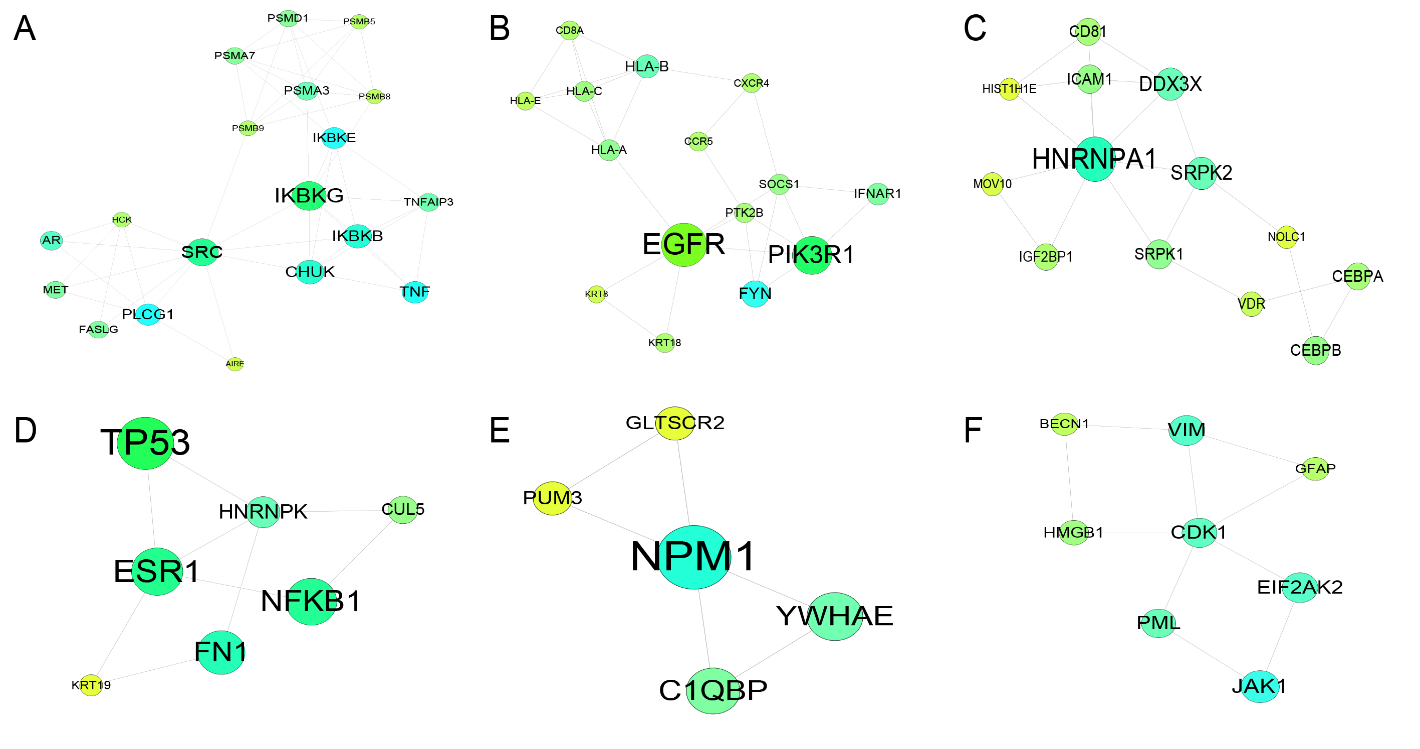


Figure S2. Results of cluster analysis for Hepatitis B disease target PPI Network (A, B, C,D,E,F stand for cluster 1, 2, 3,4,5,6)


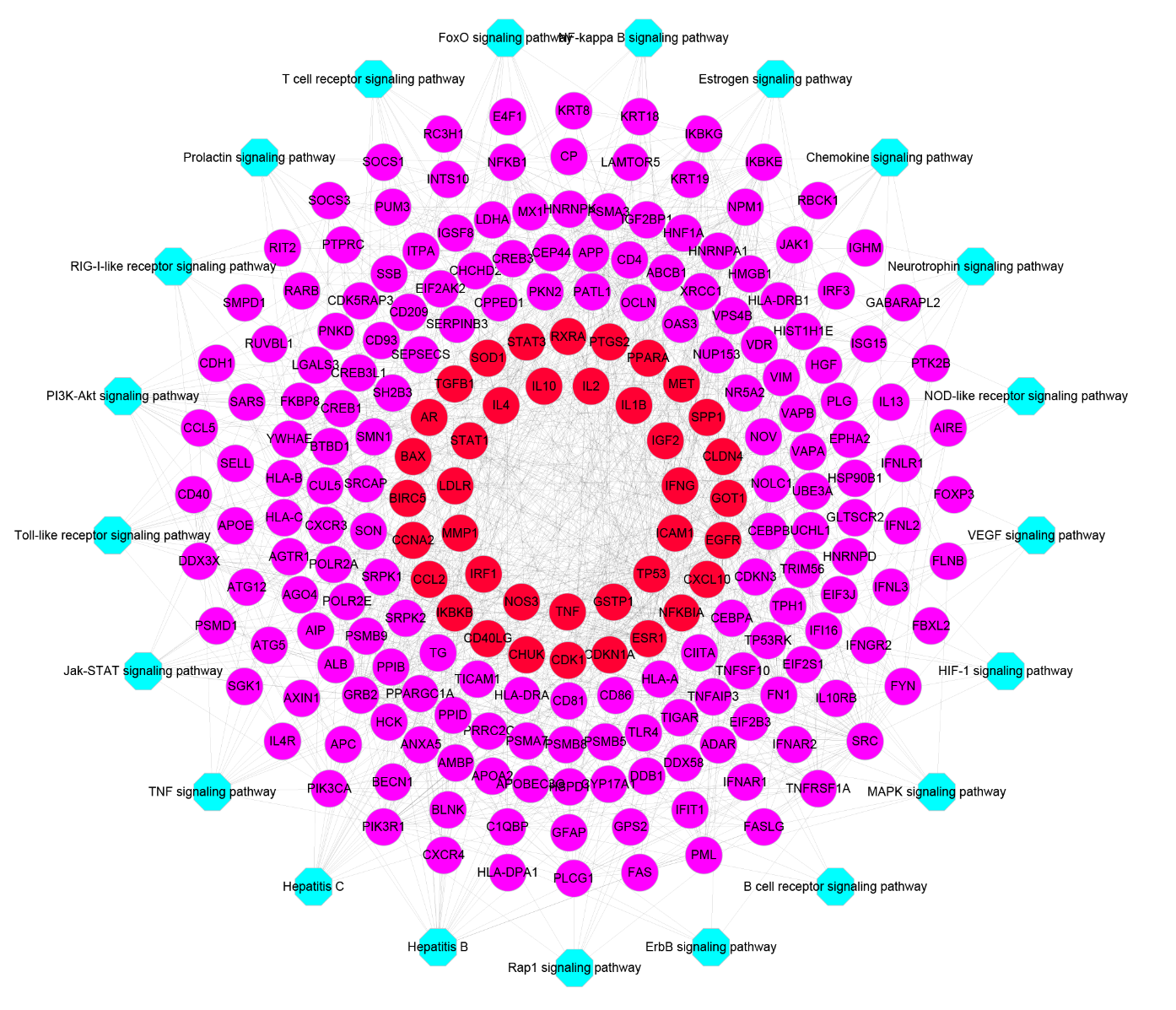


Figure S3. Pathway of YGL-Hepatitis B (The red circle represents the direct target of YGL capsule, the magenta represents the indirect target, and the blue square represents the pathway..)
